# Equine Stomach Development in the Foetal Period of Prenatal Life—An Immunohistochemical Study

**DOI:** 10.3390/ani13010161

**Published:** 2022-12-31

**Authors:** Dominik Poradowski, Aleksander Chrószcz

**Affiliations:** Department of Animal Physiology and Biostructure, Division of Animal Anatomy, Faculty of Veterinary Medicine, Wrocław University of Environmental and Life Sciences, Kożuchowska 1, 51-631 Wrocław, Poland

**Keywords:** horse, stomach, development, foetal period, APUD cells, immunohistochemistry

## Abstract

**Simple Summary:**

The endocrine cells of the alimentary tract (APUD) are an important component of the mucosa structure and significantly influence the stomach and intestine physiology. Prenatal development of the stomach is not limited to the qualitative and quantitative changes observed in the gastric wall, but is also reflected in APUD cell occurrence and their potential function in the development and maturation of the organ. Moreover, the perinatal period, crucial for the colostral globulin intake by the new-born animal, requires an adequately prepared gastric mucosa and its excretory function must be controlled. This study was aimed at immunohistochemical changes observed in the foetal period in equine foetuses. The changes were related to the reactivity of the endocrine cells and allowed us to better understand the developmental processes taking place both in the prenatal and perinatal period.

**Abstract:**

The study consisted of the immunohistochemical analysis of fundic and pyloric mucosa in the equine stomach between the 4th and 11th month of gestation. The accessible material was classified into three age groups using the CRL method. The adult reference group was used to define potential differences between foetal and adult populations of gastric APUD cells. The samples were preserved, prepared, and stained according to the standard protocols. The immunohistochemical reaction was assessed using the semi-quantitative IRS method. The results were documented and statistically analysed. The most significant increase was seen in gastrin (G) cell activity. The activity of other endocrine cells (cholecystokinin (I) cells, somatostatin (D) cells, and somatotropin receptor (SR) cells) was less dynamic. This study proved that the development of APUD cells within the stomach mucosa undergoes quantitative and qualitative changes during stomach development. Our results correspond with the findings described in the accessible literature and prove a strong correlation between morphological changes in the stomach wall and the organ development, growth, and maturation.

## 1. Introduction

The classical comparative anatomy classified the equine stomach as intermediate between the monogastric simple stomach of carnivores and the polygastric complex stomach of ruminants [1]. A detailed structure of the stomach in adult animals is well known. The organ can be divided into three basic parts (cardiac part, body of stomach, and pyloric part). The largest morphological changes can be observed in the stomach of herbivores in which the prominent transformed proventricular part forms the poligastric stomach in ruminants and blind ventricular sac in horses. The border between the non-glandular and glandular part of the equine stomach is the placated edge margin. Endocrine cells were reported in the mucous membrane of the stomach body and the pyloric part [2]. The alimentary tract embryology studies used to be aimed at the embryogenesis and the embryonic period of organogenesis, and referred to the morphological changes of the stomach during the foetal period only in general [3,4,5,6]. The majority of scientific interest has been devoted to ruminants and swine [7,8,9,10,11,12,13,14], and papers aimed at the equine embryology are rare [15,16,17,18]. Additionally, laboratory animals were frequently used as models for the investigations of gastrointestinal tract prenatal development [11,19,20,21,22,23,24,25,26,27]. Finally, the detailed description of the swine stomach prenatal development together with the studies on the organ topography, vascularisation, and innervation in the foetal period carried out by Chrószcz [28,29,30], encouraged us to investigate the equine stomach development in the foetal period [17,18]. Poradowski and Chrószcz [17,18] proved that the stomach mucosa development and maturation occurred not only in the prenatal period but continued also after birth. This work is the third part of this project (N060/0016/21—“Morphology and development of equine stomach wall (*Eqqus caballus*) in foetal period”), aimed at filling the gaps in our knowledge on the endocrine cells (APUD) development in the equine gastric glands during the foetal period.

The term APUD (amine precursor uptake and decarboxylation) was introduced by Pearse and Takor [31], and quickly became of scientific interest to many researchers [32,33,34,35,36,37,38,39,40,41]. The immunochemistry of the equine respiratory tract APUD cells was studied as a rare example of works devoted to this species and the field of research [42]. The prenatal and postnatal development of APUD cells was elaborated on in sheep [43], and a study on the swine alimentary tract in a similar context of APUD immunohistochemistry was carried out [37]. Therefore, a lack of any studies on prenatal development of APUD cells in equine stomach encouraged us to investigate this topic in detail.

The stimulating and modulating role of APUD cells is crucial for the normal physiological function of the stomach. Apart from the well-known clinical and physiological roles of APUD cells, their postnatal existence in the gastric and intestinal mucosa shall be compared with their occurrence and functions during the prenatal development of the alimentary tract. Earlier papers described the topography and morphology of equine stomach, including its histology and gland histochemistry [17,18]. While a variety of APUD cells have been identified within the gastrointestinal mucosa [44], we decided to investigate the cells most important for the gastric gland activity. Our earlier studies focused on the prenatal development of the gastric wall and subsequent occurrence of the chief and parietal cells crucial for the stomach mucosa excretory function. In this study, we examined the endocrine cells containing somatostatin (D cells), cholecystokinin (I cells), gastrin (G cells), and secretin-receptor (SR cells). Even though the literature contains information also on serotonin/5-HT(EC cells), histamine (ECL cells), and pancreatic polypeptide (PP cells), as reported by Fawcett [45] and Dellmann and Eurell [46], we investigated the immunohistochemical characteristics of only the most important APUD cells within the wall of the developing stomach in the foetal period. The aim of this study was to describe the qualitative and quantitative changes in APUD cell population in developing stomach mucosa in the foetal period. The lack of any information in this field, not only in horses, encouraged us to carry out this project.

## 2. Materials and Methods

The accessible material consisted of 20 foetuses of the Wielkopolski horse breed of a known age verified on the basis of CRL as ranging from the 4th to the 11th month of pregnancy. All specimens belonged to the collection of the Division of Animal Anatomy (Wrocław University of Environmental and Life Sciences). The foetal material was gathered and preserved ex tempore according to a standard protocol. Similarly, the slides were prepared and the immunohistochemical reaction was carried out afterwards. All mares were healthy and the foetuses did not show any malformations. The foetuses from which the study material was collected were divided into three age groups (n = 5) using the population cross-section method [17,18,47]:

- The first age group (4th–5th month of gestation).
- The second age group (7th–8th month of gestation).
- The third age group (10th–11th month of gestation).

The adult reference group was an assemblage of fully developed stomachs coming from 5–8-year-old animals, which were taken from a slaughterhouse (the fundic and pyloric part from each of the five horses). All animals were classified as healthy during the pre-slaughter examination carried out by a veterinarian. According to Polish law, the tissue sample acquisition from the cadavers or slaughtered animals did not require the permission of the ethics committee.

The collected rectangular (1 cm^2^ in size) samples of gastric mucosa from the fundic and pyloric part of the stomach were preserved in 4% buffered paraformaldehyde solution quantum satis according to a standard protocol [18]. The samples were taken from the body of the stomach and the pyloric part, as these are the locations where the largest accumulation of APUD cells has been reported in adult animals.

### 2.1. Immunohistochemical Staining

The fragments of stomach wall were fixed in 4% neutrally buffered formaldehyde, dehydrated in alcohol series, cleared in xylene, and embedded in paraffin. The paraffin blocks were sliced with the Micron HM310 microtome (Thermo Fisher Scientific, Waltham, MA, USA) into 5 μm sections. The sections were stained with hematoxylin and eosin (H&E) (Sigma-Aldrich, Saint Louise, MO, USA, cat. No. MHS32-1L and HT110232-1L), and the immunohistochemical analysis was carried out. Primary rabbit antibodies (Table 1) were used.

**Figure 1:**
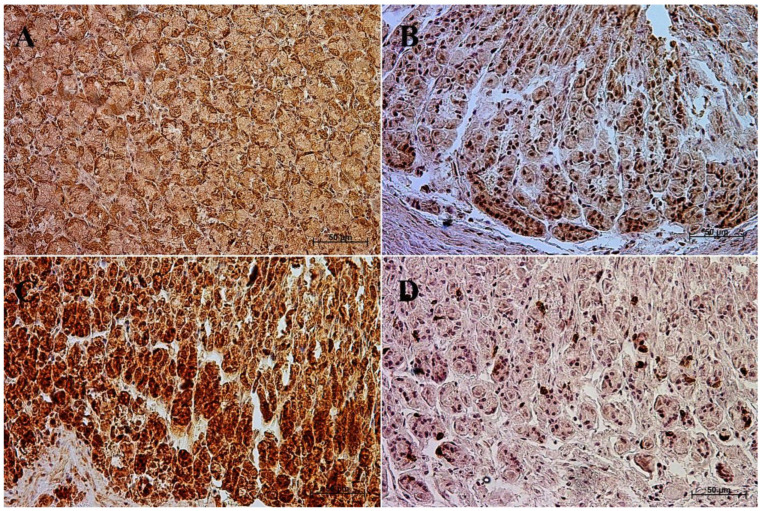
Positive control reaction for each cell marker (murine gastric mucosa), 40×. (**A**) gastrin, (**B**) cholecystokinin, (**C**) secretin receptors, (**D**) somatostatin.

**Figure 2:**
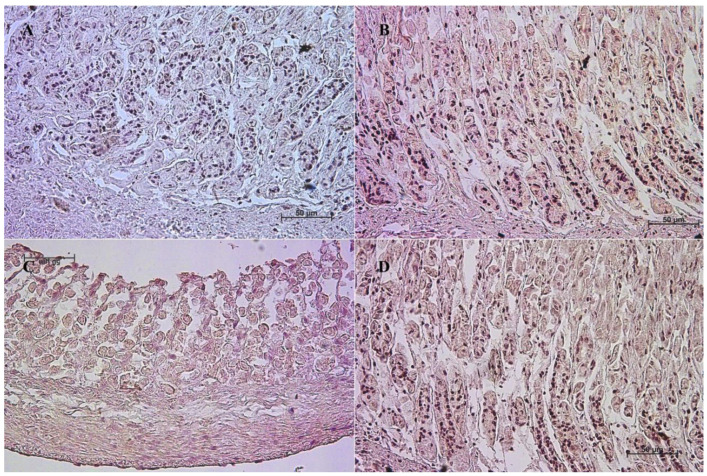
Negative control reaction for each cell marker (murine gastric mucosa), 40×. (**A**) gastrin, (**B**) cholecystokinin, (**C**) secretin receptors, (**D**) somatostatin.

The primary antibodies were detected using ImmPRESS REAGENT KIT anti-rabbit IgG according to the manufacturer’s instructions (Vector, Burlingame, CA, USA). The antigen retrieval was performed using a sodium citrate buffer, pH 6 (for anti-somatostatin antibody), and Tris/EDTA buffer, pH 9 (for anti-gastrin, anti-secretin, and anti-cholecystokinin antibodies), for 20 min at 97 °C in a water bath. The slides were incubated in a 3% H_2_O_2_ solution to quench the endogenous peroxidase activity and blocked in a 2.5% normal goat blocking serum. The sections were incubated for 1 h at room temperature with anti-somatostatin antibody, and for the night with anti-secretin receptor antibody, anti-cholecystokinin antibody, and anti-gastrin antibody at 4 °C. ImmPACT™ DAB (Vector, Burlingame, CA, USA) was used as a chromogen. The slides were counterstained with Meyer’s Hematoxylin (Sigma-Aldrich, Saint Louise, MO, USA). Positive (with the antibodies described in the protocol) and negative (without the antibodies) controls were performed for each cell marker—positive (Figure 1) and negative (Figure 2).

### 2.2. Immunohistochemical Reaction Scoring

Evaluation of the expression of somatostatin, gastrin, cholecystokinin, and secretin receptors was carried out using the semi-quantitative IRS method according to the Remmele scale [48,49]. This method estimates the percentage of cells showing a positive response (I) and evaluates their intensity (II) (Table 2) according to formula I × II. The assessment was performed using a light microscope (Zeiss Axio Scope A1; Carl Zeiss Jena, Germany) in five fields of view at 40× magnification by two independent researchers experienced in the evaluation of immunohistochemical reactions.

### 2.3. Statistical Analysis

For the statistical analysis, the OriginPro package, version 2021 (OriginLab Corporation, Northampton, MA, USA) was used. The data obtained for individual parameters were averaged and the standard deviation (SD) was calculated. The regression curves were made using the Simple Fit application in OriginPro. Based on the results, plotted on the diagrams, nonlinear regression curves (polynomial approximation) and correlation between the variables were obtained. All the results are shown in tables and diagrams.

## 3. Results

The study outcomes were divided according to the foetus’s age and the part of the stomach mucosa in which the immunohistochemical reactions were performed.

### 3.1. Immunohistochemistry of Anti-Gastrin Antibody

**Figure 3:**
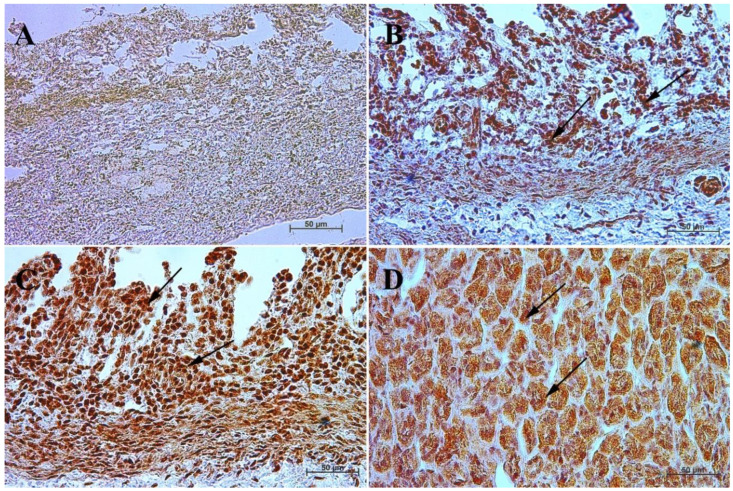
Immunoreactivity of anti-gastrin antibody in the fundic mucosa of the stomach, 40×. (**A**) the first age group, (**B**) the second age group, (**C**) the third age group, (**D**) adult reference group. The arrow indicates positive immunohistochemical reaction.

**Figure 4:**
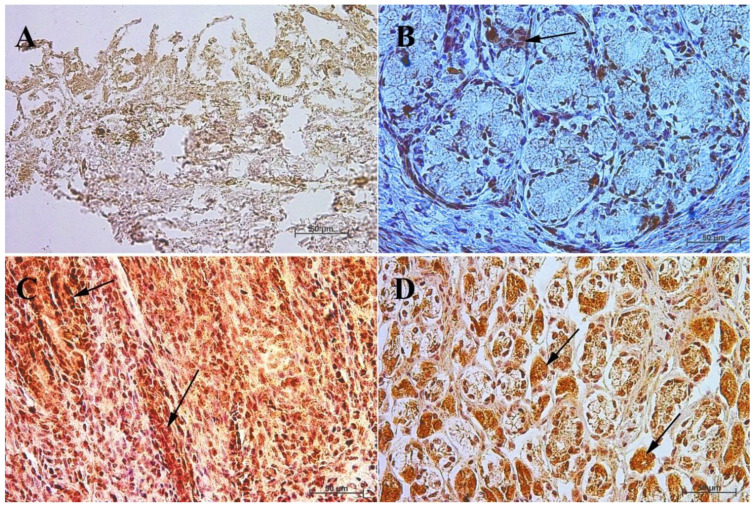
Immunoreactivity of anti-gastrin antibody in the pyloric mucosa of the stomach, 40×. (**A**) the first age group, (**B**) the second age group, (**C**) the third age group, (**D**) adult reference group. The arrow indicates positive immunohistochemical reaction.

In the first and second age group, a moderate cytoplasmic reaction of potential precursors of G cells was observed in the fundus of the stomach mucosa, and a mild immunohistochemical reaction was seen in the pyloric region. This was in contrast to the third group, where in both tested parts of the stomach the reaction was strong, and similar to that observed in adult horses (Figure 3 and Figure 4, Table 3).

### 3.2. Immunohistochemistry of Anti-Cholecystokinin Antibody

**Figure 5:**
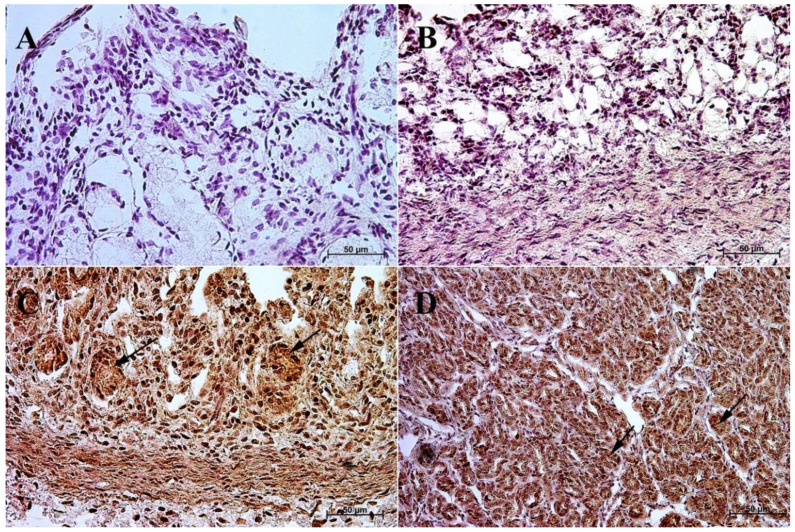
Immunoreactivity of anti-cholecystokinin antibody in the fundic mucosa of the stomach, 40×. (**A**) the first age group, (**B**) the second age group, (**C**) the third age group, (**D**) adult reference group. The arrow indicates positive immunohistochemical reaction.

**Figure 6:**
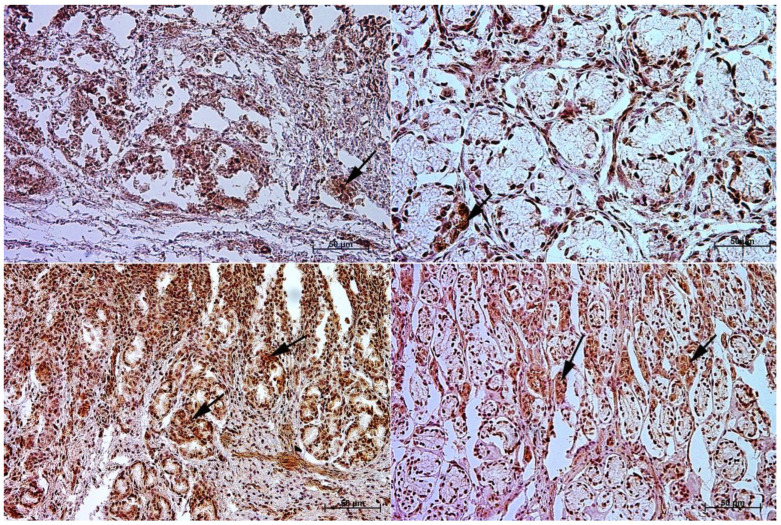
Immunoreactivity of anti-cholecystokinin antibody in the pyloric mucosa of the stomach, 40×. (**A**) the first age group, (**B**) the second age group, (**C**) the third age group, (**D**) adult reference group. The arrow indicates positive immunohistochemical reaction.

In the case of cholecystokinin, the lack of its expression in the gastric fundus in both the first and the second age group was noted, while in the pyloric part of the stomach the potential precursors of I cells were identified, and the immunohistochemical reaction was mild in the first group and moderate in the second group. In the third group, a strong cytoplasmic reaction was observed both in the fundic and the pyloric parts of the stomach. In the reference group of adult horses, the strong reaction remained visible only in the fundic mucosa of the stomach, while in the pylorus it was weakened (moderate) (Figure 5 and Figure 6, Table 4).

### 3.3. Immunohistochemistry of Anti-Secretin Receptor Antibody

**Figure 7:**
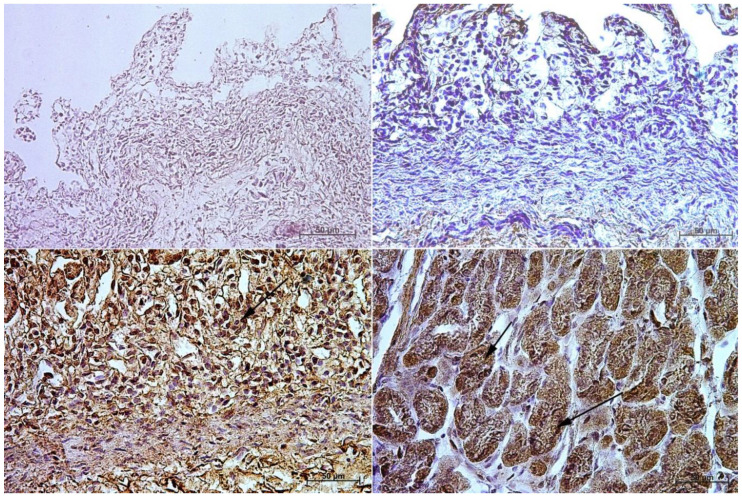
Immunoreactivity of anti-secretin receptor antibody in the fundic mucosa, 40×. (**A**)—the first age group, (**B**)—the second age group, (**C**)—the third age group, (**D**)—adult reference group. The arrow indicates positive immunohistochemical reaction.

**Figure 8:**
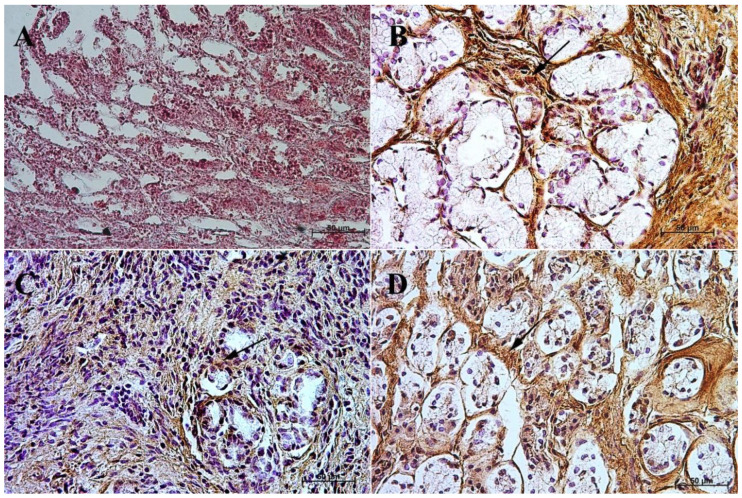
Immunoreactivity of anti-secretin receptor antibody in the pyloric mucosa, 40×. (**A**) the first age group, (**B**) the second age group, (**C**) the third age group, (**D**) adult reference group. The arrow indicates positive immunohistochemical reaction.

The expression of secretin receptors (SR cells and their potential precursors) in the first two studied groups was negative in the fundic region and mild in the pyloric part. In the third group, the expression in the fundic mucosa increased, while in the pyloric part it remained at the same level. In adult horses, as compared with the three previous groups (Figure 7 and Figure 8, Table 5), the expression of the studied receptors increased, which clearly proved that secretin receptors are most intensely expressed only after birth.

### 3.4. Immunohistochemistry of Anti-Somatostatin Antibody

**Figure 9:**
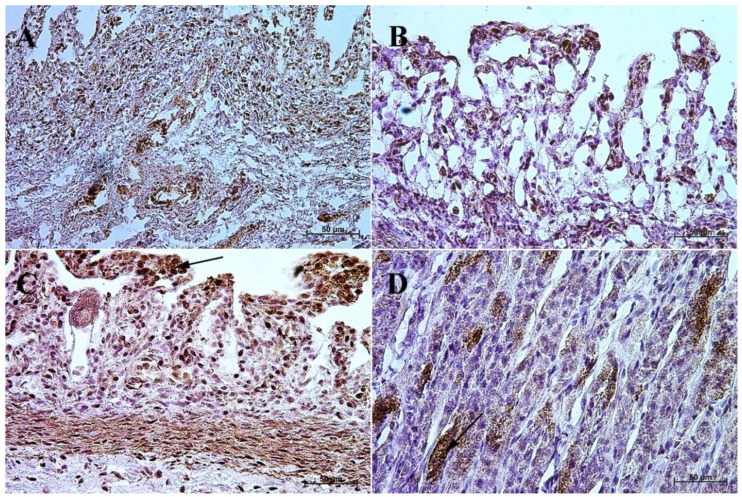
Immunoreactivity of anti-somatostatin antibody in the fundic mucosa, 40×. (**A**) the first age group, (**B**) the second age group, (**C**) the third age group, (**D**) adult reference group. The arrow indicates positive immunohistochemical reaction.

**Figure 10:**
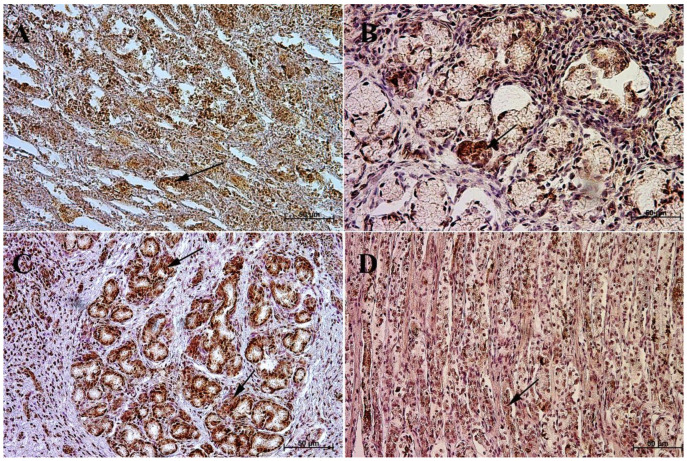
Immunoreactivity of anti-somatostatin antibody in the pyloric mucosa, 40×. (**A**) the first age group, (**B**) the second age group, (**C**) the third age group, (**D**) adult reference group. The arrow indicates positive immunohistochemical reaction.

In the first and second age group, the intensity of the cytoplasmic reaction to the anti-somatostatin antibody in the potential precursors of D cells was mild in the fundic part of the stomach and moderate in the pylorus. In the third group, the reaction in both gastric wall regions was intensified and looked the same as in the group of adult animals, i.e., moderate in the fundic and strong in the pyloric part of the stomach mucosa (Figure 9 and Figure 10, Table 6).

Statistical analysis of the results showed significant differences between each of the three age groups and the adult reference group. Earlier studies by Poradowski and Chrószcz [17,18] proved that the equine foetal crown rump length (CRL) can be described as isomeric, as the statistical analysis of CRL values in the foetal period indicated a linear correlation with the foetus age. A comparison of the gastric morphometric and histometric values with the CRL allowed for a detailed description of the growth rate dynamics taking into consideration the investigated parameters [17,18]. A similar methodology was used in this paper.

**Figure 11:**
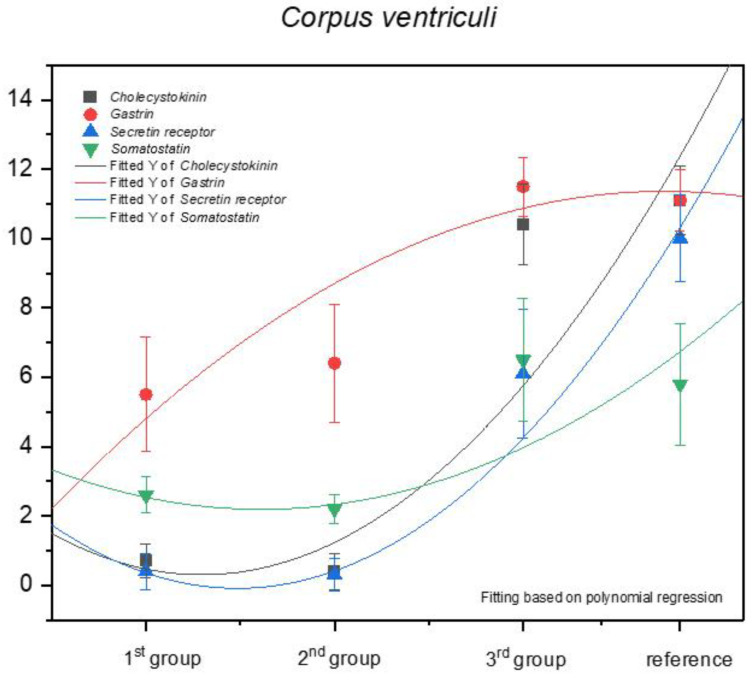
Expression of selected antibodies in the stomach of the investigated age groups.

Statistical analysis of the results for the fundic mucosa proved that the increase in the positive anti-gastrin immunohistochemical reaction can be described as positive allometric, and the quantity of G cells in the third age group and in the adult reference group was comparable (Figure 11).

Contrary to the above mentioned findings, the immunohistochemical reaction for the other antibodies (anti-cholecystokinin, anti-secretin receptors, and anti-somatostatin) must be classified as negative allometric, especially in the first and second age group, approaching isometric in the third age group and the adult reference group. The regression curves estimated for cholecystokinin and secretin receptor expression were similar to each other, and the one for somatostatin showed lower intensity.

**Figure 12:**
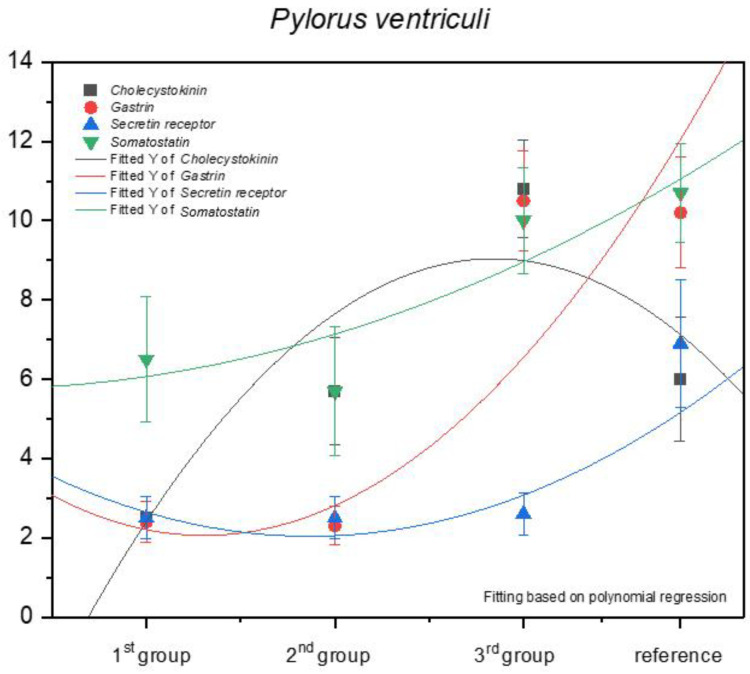
Expression of selected antibodies in the pyloric part of the stomach in the investigated age groups.

The increase in the expression of somatostatin was similar in the pyloric and fundic part of the stomach (negative allometric). The immunohistochemical reaction of the anti-secretin receptors showed a negative allometric increase only in the third age group and the adult reference group (Figure 12).

The strongest but still negative allometric increase was observed for the anti-gastrin reaction when comparing the second and the third age groups. The expression of this antibody in the third age group and the reference group was comparable. Finally, a positive allometric increase in anti-cholecystokinin antibodies was visible in the foetal period. A comparison of the third age group and the adult reference group indicated an intense decrease in the positive immunohistochemical reaction in the pyloric region.

## 4. Discussion

The endocrine cells (APUD cells) accumulate within the epithelium of the alimentary tract mucosa, especially in the pyloric part of the stomach and the duodenum [33,34,35,36,37,38,40,41,42,43,50]. The hormones produced by these cells can be described as equivalents of neurotransmitters and the activity of these cells is controlled by physical and chemical stimuli, including the composition of the gut lumen chyme [34,51]. The enteric hormones can influence the environment directly (paracrine route) or can exert their effect in the target organs (endocrine route) [34,41]. The APUD cells are classified into 12 types based on their hormonal activity [50]. The traditional classification of APUD cells is still valid, but the same cells can produce more than one substance (e.g., I cells—serotonin, GIP, PYY, and ghrelin) [52,53]. Recent studies reported also the presence of neurotransmitters and neuropeptides in the endocrine cells of the alimentary tract [53]. Finally, the function of the APUD cells is linked with the physiology of the enteric nervous system cells, which are capable of receiving stimuli from the endocrine and paracrine routes [54]. All nervous cells of the enteric nervous system develop from progenitors migrating from the neural crest to the wall of the alimentary tract [51,55]. Detailed studies on the development of the human enteric nervous system proved that the process is not finished at the moment of birth but continues during childhood and is stabilized during adolescence [56,57,58]. Therefore, the description of APUD cell activity in the mucosa of the equine stomach during the prenatal life, together with the observations made in adult animals, may shed some light on the still unknown changes occurring in the gastric endocrine cell populations in horses. The gastric acid secretion and synthesis and the secretion of gastrin play an important role in the normally developing stomach. Moreover, the acid secretory capacity of the stomach is important to prevent detrimental microbial activity and to control the activity of gastric proteolytic enzymes [36]. In pigs, basal acid secretion starts in the prenatal life [59]. Gastrin and somatostatin are known as the most important peptides regulating the function of fundic parietal cells [60]. Finally, in humans, there is a positive correlation between the parietal cell density and maximal acid output, and a negative correlation between the parietal cell density and the patient’s age. Moreover, a significant positive correlation was found between the G cell mass and basal acid output. Parietal cell density and maximal acid output play an important role in the gastric cancer and duodenal ulcers [32]. Gastric ulcers are a well-known condition in horses and the disease is an important economic factor in the animal breeding and use [61]. Therefore, a detailed description of the stomach development, including the identification of changes in the APUD cell populations in prenatal life, can be a valuable contribution not only to morphological but also to clinical research. This is especially interesting, as an important role of gastrointestinal endocrine cells in human pathologies has also been proven [62].

The secretin receptor expression within the gastric mucosa is important for the physiological regulation of the gastric gland secretion. The duodenal mucosa (S cells) produces a strong inhibitor of gastrin release (G cells) and gastric acid secretion (parietal cells) responding to the presence of food in the duodenum. Cholecystokinin can also significantly inhibit the gastrin-mediated acid release due to its competition, antagonism, and binding to the CCK-B receptors on the oxyntic cells or potent antagonism of gastrin-stimulated acid secretion due to CCK-B receptors on D cells (somatostatin inhibits acid secretion) [63]. Therefore, the occurrence and immunohistochemical identification of potential APUD cell precursors and anti-secretin receptors in the foetal gastric mucosa seem important for better a understanding of the gastric wall development and function in the prenatal and perinatal period. Some information was provided by the studies on the prenatal development of swine alimentary tract and the immunohistochemical characterisation of the gastric, duodenal, and pancreatic endocrine cells [37]. However, the accessible literature does not address this topic regarding the equine foetal period.

Earlier studies by Poradowski and Chrószcz [17,18] demonstrated dynamic changes in the stomach morphology and histology. The most important parts of the gastric mucosa associated with the occurrence of the endocrine cells in prenatal life are the pyloric and fundic regions. The mucosa of the glandular part of the stomach developed from a single columnar epithelium forming the gastric pits and the mesenchyme with a small number of blood vessels forming *lamina propria mucosae* in the first age group. The secondary differentiation into three basic gastric parts: cardiac, fundic, and pyloric mucosa, visible parietal cells within the wall of the fundic glands, loose connective tissue in *lamina propria mucosae*, and well distinguished myocytes of *lamina propria mucosae* were observed in the second age group. Finally, in the third age group, the gastric glands were classified as tubular glands penetrating the stroma of the connective tissue and forming large glandular complexes, especially in the fundic (with the chief and parietal cells) and pyloric parts of the gastric mucosa [17,18]. This process seems strongly linked to the occurrence and immunohistochemical identification of cells playing important roles in endocrine and paracrine activity of the developing stomach mucosa (Figure 3, Figure 4, Figure 5, Figure 6, Figure 7, Figure 8, Figure 9 and Figure 10, Table 2, Table 3, Table 4, Table 5 and Table 6). Our results demonstrated a sudden increase in gastrin production between 7–8 and 10–11 months of gestation (Figure 11 and Figure 12). Increasing population of gastrin-positive immunoreactive cells during prenatal development (starting form 142 day of gestation) was also observed in red deer embryological studies [64]. Moreover, similar studies on the prenatal development of rabbit, aimed at somatostatin and gastrin regulation in the gastric acid production, revealed that decreasing the somatostatin-to-gastrin ratio in advanced pregnancy enhanced the foetal gastric acid secretory activity in the fundic mucosa [65]. This may indicate a rapid development of G cells and the resulting intensification of gastrin staining. This is probably intended to prepare the stomach for food intake. The morphological and histological studies of the stomach in the equine foetal period also indicated the most important developmental changes occurring in late pregnancy [17,18]. Moreover, the well-known process of the stomach mucosa maturation occurs in the late foetal and perinatal period, and it serves as the crucial point of the functional start not only for the stomach activity, but also for the new-born animal and the colostrum globulin reception [5,14,27,28,29,30].

Cholecystokinin was not expressed in the fundic region in the first and second group, while the I cell precursors were identified in the pyloric region even in the first age group, and more clearly in the second age group. A strong cytoplasmic reaction in both gastric mucosa regions was also observed in the third group (remaining only in the fundic mucosa in the adult reference group). All these can be explained by the fact that cholecystokinin secretion was taken over by the duodenum, and its presence in both investigated parts of the gastric mucosa proves its function (inhibition of gastric peristalsis) was restricted but still maintained in the gastric mucosa. Simultaneously, the increasing amount of secretin receptors, depending on the age of the foetus (Figure 11 and Figure 12), signalled the preparatory processes aimed at increasing the secretion in the stomach and intestines, and thus, to prepare the new-born animal for food intake. Somatostatin expression is definitely lower in the fundic part of the stomach than in the pylorus (Figure 11 and Figure 12). This is presumably due to the fact that somatostatin secreted into the lumen of the duodenum more easily penetrates the cells of the gastric pyloric wall than the fundic wall. This fact may also indicate a progressive development of pancreatic D cells, which, through the production of somatostatin, affect, among others, the production of gastric juice.

Statistical analysis of our earlier results demonstrated positive allometric growth of the fundic gastric mucosa and fundic glands, which indicated the greatest role of these glands’ growth in the thickness of the stomach wall, as compared with the cardiac and pyloric glands [17,18]. Taking into consideration the dynamic changes in the expression of the investigated antibodies (anti-gastrin, anti-cholecystokinin, anti-somatostatin, and anti-secretin receptors), the positive allometric increase of their expression was found in the cells reacting to anti-gastrin antibody in the fundic part of the stomach and anti-cholecystokinin antibody in the pyloric region. The immunohistochemical reaction caused by the other antibodies both in the fundic and pyloric part of the gastric mucosa was negative allometric. The quantity of endocrine cells increased towards the end of the prenatal life. The number of G cells increased in a positive allometric way, similar to the growth rate of developing fundic mucosa. It can be, therefore, stated that these processes strongly correlate with each other. The other populations of the endocrine cells proliferated more slowly (negative allometric growth rate), except for the I cells in the pyloric region. The APUD cells play an important role in modulating the stomach exocrine activity and this process starts already in the prenatal period.

Finally, a comparison of the quantity of the investigated APUD cells in the stomach mucosa between the third age group and the adult reference group showed that their proliferation is not finished at the moment of birth. The alimentary tract mucosa, including the stomach, of a new-born animal plays a crucial role in the production of colostral maternal globulins and possible animal vaccination strategies [66]. A comparison of the stomach’s histological structure and metric parameters in the third group and the adult reference group demonstrated significant anatomical and histological differences [17,18]. The organ development continues in the perinatal period [18,43]. Even though the general anatomical partition of the stomach in a new-born and adult animals is similar [17], and the occurrence of glandular cell types is analogous, the most important differences were found in the values of the metric parameters, which were lower in the adult reference group than in the third age group. The parameter most important for the stomach wall thickness increase and the organ postnatal function is the positive allometric growth of the fundic mucosa and external muscular layer [18]. Even though G and D cells’ expression in the gastric mucosa is similar in the third age group and the adult reference group, the two remaining APUD cell types showed significant quantitative differences. The quantity of the I cells in the pyloric region decreased and in the fundic regions it remained comparable in both groups, whereas the SR cells population increased in both gastric mucosa regions. These changes can be linked to the physiological maturation of the organ and its adaptation to normal digestive function after the end of the perinatal period.

## 5. Conclusions

In summary, the expression of all the investigated antibodies in the stomach fundic and pyloric mucosa showed significant quantitative changes during the stomach development in the foetal period. The stimulating role of G and I cells, and the modulating role of D and SR cells have their roots in the prenatal life. Further studies on the changes in APUD cell populations in the alimentary tract are needed, especially in duodenal mucosa. Due to their endocrine and paracrine activity, the duodenal mucosa and the gastric mucosa form a joint functional unit that influences the physiological activity of both organs. Earlier studies on stomach prenatal development in the foetal period supported the thesis that the anatomical and histological changes are not limited to the prenatal organ development. All obtained data seem to prove that the foetal period of the prenatal life is important not only for the morphology and physiology of the developing stomach, but can shed light on the full understanding of processes taking place postnatally in gastric pathologies, especially important in horses.

## Figures and Tables

**Table 1:** Specification of the primary antibodies used.

Name	Type	Dilution	Catalogue Number	Homology
Gastrin	Polyclonal	1:500	clone ab16035, Abcam, Cambridge, UK	>75%
Cholecystokinin	Polyclonal	1:300	clone PA5-103116, Invitrogen, Waltham, MA, USA	unknown
Secretin receptors	Polyclonal	1:200	clone bs-0089R, Biossusa, Woburn, MA, USA	unknown
Somatostatin	Polyclonal	1:500	clone ab108456, Abcam, Cambridge, UK	>75%

**Table 2:** Immunoreactive score—IRS [47].

I—% of Positive Cells	II—Intensity of Staining	IRS Score
0 = lack	0 = lack of colour reaction	0–1 = negative
1 = <10%	1 = mild	2–3 = mild
2 = 10–50%	2 = moderate	4–8 = moderate
3 = 51–80%	3 = intense	9–12 = strongly positive
4 = >80%		

**Table 3:** Immunoreactivity of anti-gastrin antibody.

**Age [Months]**	**The first age group (4–5 Month of gestation)**											
											**IRS mean/SD**	**Description**
*Corpus ventriculi*	7	7	6	4	4	4	8	7	4	4	5.5 ± 1.65	moderate
*Pylorus ventriculi*	3	3	2	3	2	2	2	2	3	2	2.4 ± 0.52	mild
**Age [months]**	**The second age group (7–8 month of gestation)**											
											**IRS mean/SD**	**Description**
*Corpus ventriculi*	8	4	4	8	4	7	7	7	7	8	6.4 ± 1.71	moderate
*Pylorus ventriculi*	2	2	2	2	3	2	2	2	3	3	2.3 ± 0.48	mild
**Age [months]**	**The third age group (10–11 month of gestation)**											
											**IRS mean/SD**	**Description**
*Corpus ventriculi*	12	12	12	12	12	10	11	12	10	12	11.5 ± 0.85	strongly positive
*Pylorus ventriculi*	11	11	10	9	9	9	10	12	12	12	10.5 ± 1.27	strongly positive
**Age [months]**	**Adult reference group**											
											**IRS mean/SD**	**Description**
*Corpus ventriculi*	10	12	12	11	11	11	12	10	10	12	11.1 ± 0.88	strongly positive
*Pylorus ventriculi*	9	10	9	9	11	12	12	12	9	9	10.2 ± 1.4	strongly positive

**Table 4:** Immunoreactivity of anti-cholecystokinin antibody.

**Age [Months]**	**The first age group (4–5 Month of gestation)**											
											**IRS mean/SD**	**Description**
*Corpus ventriculi*	1	1	1	1	0	1	1	0	0	1	0.7 ± 0.48	negative
*Pylorus ventriculi*	2	3	3	3	2	2	3	2	3	2	2.5 ± 0.53	mild
**Age [months]**	**The second age group (7–8 month of gestation)**											
											**IRS mean/SD**	**Description**
*Corpus ventriculi*	0	0	0	1	0	1	1	0	0	1	0.4 ± 0.52	negative
*Pylorus ventriculi*	5	5	8	4	6	6	7	5	4	7	5.7 ± 1.34	moderate
**Age [months]**	**The third age group (10–11 month of gestation)**											
											**IRS mean/SD**	**Description**
*Corpus ventriculi*	9	11	9	12	12	11	11	10	9	10	10.4 ± 1.17	strongly positive
*Pylorus ventriculi*	11	11	10	9	9	12	12	12	12	10	10.8 ± 1.23	strongly positive
**Age [months]**	**Adult reference group**											
											**IRS mean/SD**	**Description**
*Corpus ventriculi*	12	12	10	12	11	11	9	12	11	11	11.1 ± 0.99	strongly positive
*Pylorus ventriculi*	6	6	8	7	7	8	6	4	4	4	6 ± 1.56	moderate

**Table 5:** Immunoreactivity of anti-secretin receptor antibody.

**Age [Months]**	**The first age group (4–5 Month of gestation)**											
											**IRS mean/SD**	**Description**
*Corpus ventriculi*	0	1	0	0	0	0	1	0	1	1	0.4 ± 0.52	negative
*Pylorus ventriculi*	2	3	2	3	2	2	3	2	3	3	2.5 ± 0.53	mild
**Age [months]**	**The second age group (7–8 month of gestation)**											
											**IRS mean/SD**	**Description**
*Corpus ventriculi*	0	0	0	0	0	1	0	0	1	1	0.3 ± 0.48	negative
*Pylorus ventriculi*	3	2	3	3	3	2	2	2	2	3	2.5 ± 0.53	mild
**Age [months]**	**The third age group (10–11 month of gestation)**											
											**IRS mean/SD**	**Description**
*Corpus ventriculi*	8	4	5	7	8	8	4	4	5	8	6.1 ± 1.85	moderate
*Pylorus ventriculi*	2	3	2	3	3	3	2	3	3	2	2.6 ± 0.52	mild
**Age [months]**	**Adult reference group**											
											**IRS mean/SD**	**Description**
*Corpus ventriculi*	11	9	9	9	9	10	10	9	12	12	10 ± 1.25	strongly positive
*Pylorus ventriculi*	8	8	8	8	8	4	5	5	8	7	6.9 ± 1.6	moderate

**Table 6:** Immunoreactivity of anti-somatostatin antibody.

**Age [Months]**	**The first age group (4–5 month of gestation)**											
											**IRS mean/SD**	**Description**
*Corpus ventriculi*	2	3	3	2	2	2	3	3	3	3	2.6 ± 0.52	mild
*Pylorus ventriculi*	8	7	8	5	5	5	8	7	8	4	6.5 ± 1.58	moderate
**Age [months]**	**The second age group (7–8 month of gestation)**											
											**IRS mean/SD**	**Description**
*Corpus ventriculi*	2	2	3	2	2	2	2	2	2	3	2.2 ± 0.42	mild
*Pylorus ventriculi*	4	7	4	4	6	6	4	6	8	8	5.7 ± 1.64	moderate
**Age [months]**	**The third age group (10–11 month of gestation)**											
											**IRS mean/SD**	**Description**
*Corpus ventriculi*	8	4	5	4	5	8	8	8	7	8	6.5 ± 1.78	moderate
*Pylorus ventriculi*	12	9	9	8	12	10	10	10	11	9	10 ± 1.33	strongly positive
**Age [months]**	**Adult reference group**											
											**IRS mean/SD**	**Description**
*Corpus ventriculi*	5	5	5	4	8	8	7	8	4	4	5.8 ± 1.75	moderate
*Pylorus ventriculi*	12	12	11	12	10	10	12	10	9	9	10.7 ± 1.25	strongly positive

## Data Availability

The data presented in this study are available on request from the first author/corresponding authors.
